# Technical challenges in generalizing calibration techniques for breast density measurements

**DOI:** 10.1002/mp.13325

**Published:** 2019-01-11

**Authors:** Erin E. E. Fowler, Autumn M. Smallwood, Nadia Z. Khan, Kaitlyn Kilpatrick, Thomas A. Sellers, John Heine

**Affiliations:** ^1^ Cancer Epidemiology Department Moffitt Cancer Center & Research Institute 12902 Bruce B. Downs Blvd Tampa FL 33612 USA; ^2^ Corporate Compliance Department Moffitt Cancer Center & Research Institute 12902 Bruce B. Downs Blvd Tampa FL 33612 USA

**Keywords:** breast density, calibration, mammography, thickness correction

## Abstract

**Purpose:**

We are developing a calibration methodology for full‐field digital mammography (FFDM). Calibration compensates for image acquisition technique influences on the pixel representation, ideally producing improved inter‐image breast density estimates. This approach relies on establishing references with rigid breast tissue‐equivalent phantoms (BTEs) and requires an accurate estimate of the compressed breast thickness because the system readout is nominal. There is also an attenuation mismatch between adipose breast tissue and the adipose BTE that was noted in our previous work. It is referred to as the “attenuation anomaly” and addressed in this report. The objectives are to evaluate methods to correct for the compressed breast thickness and compensate for the attenuation anomaly.

**Methods:**

Thickness correction surfaces were established with a deformable phantom (DP) using both image and physical measurements for three direct x‐ray conversion FFDM units. The Cumulative Sum serial quality control procedure was established to ensure the thickness correction measurements were stable over time by imaging and calibrating DPs biweekly in lieu of physical measurements. The attenuation anomaly was addressed by evaluating adipose image regions coupled with an optimization technique to adjust the adipose calibration data. We compared calibration consistency across matched left and right cranial caudal (CC) mammographic views (n = 199) with and without corrections using Bland–Altman plots. These plots were complemented by comparing the right and left breast calibrated average (μ_a_) and population distribution mean (m_a_) with 95% confidence intervals and difference distribution variances with the F‐test for uncorrected and corrected data.

**Results:**

Thickness correction surfaces were well approximated as tilted planes and were dependent upon compression force. A correction was developed for the attenuation anomaly. All paddles (large and small paddles for all units) exhibited similar tilt as a function of force. Without correction, m_a_ = 0.92 (−1.77, 3.62) was not significantly different from zero with many negative μ_a_ samples. The thickness correction produced a significant shift in the μ_a_ distribution in the positive direction with m_a_ = 13.99 (11.17, 16.80) and reduced the difference distribution variance significantly (*P *<* *0.0001). Applying both corrections in tandem gave m_a_ = 22.83 (20.32, 25.34), representing another significant positive shift in comparison with the thickness correction in isolation. Thickness corrections were stable over approximately a 2‐year timeframe for all units.

**Conclusion:**

These correction techniques are valid approaches for addressing technical problems with calibration that relies on reference phantoms. The efficacy of the calibration methodology will require validation with clinical endpoints in future studies.

## 1 Introduction

Breast density is a strong breast cancer risk factor, typically assessed from two‐dimensional (2D) mammograms. Women with an extensive degree of breast density have a two to sixfold higher risk of breast cancer compared to women with little density.^1^, ^2^, ^3^, ^4^ Although there are several established methods to analyze mammograms for risk purposes,^3^, ^5^, ^6^, ^7^, ^8^ to date there is not a recognized standard for breast density determination clinically.^9^ To address this situation, our ongoing breast density work includes developing calibrated metrics based on imaging reference phantoms. Calibration compensates for the x‐ray image acquisition technique influences on the pixel representation, ideally producing inter‐image normalization and improved breast density estimates. Our focus is specific to techniques that use breast tissue equivalent (BTE) phantoms to establish references. In theory, calibration should produce a more accurate breast density measurement. To date, the improved precision achieved using phantom‐based calibration with 2D mammography has been mixed, although promising, in their associations with breast cancer.^5^, ^6^, ^8^, ^10^, ^11^, ^12^

We are currently modifying a calibration methodology developed previously.^13^, ^14^, ^15^ In this prior work, we developed a phantom‐based calibration approach using a specific indirect x‐ray detection full‐field digital mammography (FFDM) unit. This technique produced several calibrated breast density metrics that provided strong associations with breast cancer.^10^, ^11^, ^12^, ^16^ Two areas were identified that could be amenable for improvement. These included estimating the compressed breast thickness across the image field of view (FOV)^14^, ^17^, ^18^, ^19^, ^20^, ^21^ and compensating for differences between the x‐ray attenuation properties of adipose breast tissue and the respective adipose BTE phantom material,^11^ referred to as the attenuation anomaly in this report. The compressed breast thickness readout (system height) provided by a given mammography unit only provides nominal accuracy and implies the compression paddle is parallel with the breast support surface, and cannot account for deviations in breast thickness due to paddle tilt and deflection. A method was devised to isolate these two effects (paddle tilt and attenuation anomaly) as their influences on calibration accuracy may be similar and difficult to separate. We present a methodology in this report that both simplifies and builds upon our previous calibration work to develop a standardized breast density measure.^14^

## 2 Materials and methods

### 2.A. Images, patient data, and processing

**Figure 1:**
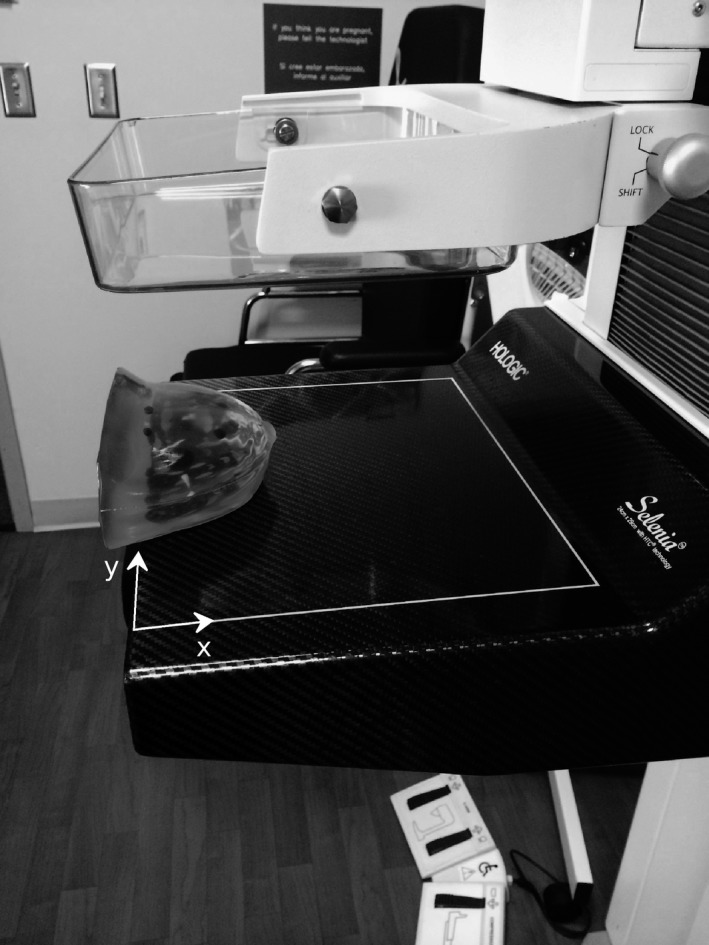
Breast Support Surface, Biopsy Practice Phantom, and Coordinate System: This phantom was used to develop the compressed breast thickness correction. The cusp (left side) was avoided by keeping it outside of the detector field of view (FOV). The positive x and y coordinate directions are indicated, the z direction is orthogonal to the support surface plane, and the origin, (0,0,0), is located on the bottom left.

Images were acquired from three Hologic Selenia^®^ FFDM units, referred to as H1, H2, and H3. These units were used for routine breast cancer screening at Moffitt Cancer Center, Tampa, FL, during the timeframe of data collection. These systems use direct x‐ray conversion^22^ and are equipped with a Fully Automatic Self‐adjusting Tilt compression paddle referred to as a FAST paddle™ (Hologic, Inc., Bedford, MA, USA). This paddle, shown in Fig. 1, is semirigid (i.e., tilts when under stress). In the screening environment, two types of FAST paddles are used differing in size. The choice of paddle is based on the x‐ray technologist's judgment, dependent upon the patient's breast size. The FOV adjusts automatically when a given paddle is attached: 24 cm × 29 cm and 18 cm × 24 cm for the large and small FOVs, respectively. We refer to Fig. 1 (see caption) to establish a coordinate system. Points on the breast support surface plane correspond to the image coordinates (x,y) in pixel units. The (x,y) coordinates range from (0,0) to (2559,3327) or (3327,4095) in the small and large FOVs, respectively. The detector has a 70‐μm pitch, and raw data are in monochrome 1 format with 14 bit per pixel dynamic range (i.e., adipose tissue is bright). Two units (H1 and H2) have Tungsten/Rhodium (W/Rh) and Tungsten/Silver (W/Ag) target/filter options. The third unit (H3) has Molybdenum/Molybdenum (Mo/Mo) and Molybdenum/Rhodium (Mo/Rh) options. Images for this study were acquired over the 25–34 kV range. We use raw images in the cranial caudal (CC) view as study images for calibration purposes to avoid pectoral muscle interference. Mammograms used in this study were from patients visiting the breast cancer screening facilities at the Moffitt Cancer Center, Tampa, FL. The dataset consists of screening mammograms from 199 women without a history of breast cancer collected under an approved IRB protocol. Mammograms were acquired from one of the three study units between July 2013 and January 2016. Image processing was performed in the IDL environment (Version 8.6, Exelis Visual Information Solutions, Boulder, CO, USA) and regression analyses using SAS (V9.4, SAS Institute Inc., Cary, NC, USA).

### 2.B. Calibration approach

The calibration methodology for mammograms was described previously.^14^, ^15^, ^23^, ^24^ Briefly, calibration maps the image data into standardized units (SUs), which is a normalized effective x‐ray attenuation representation, ranging from (0,100). The attenuation anomaly will induce negative SUs, whereas inaccurate compressed breast thicknesses can induce inaccurate calibration in both directions. The lower and upper SU range represents adipose and extremely dense tissue, respectively. We used the mean calibrated pixel quantity (defined as μ) from a given mammogram (or phantom) as the measure of breast density in this report because of its established relationship with breast cancer.^10^ We define the logarithmic relative exposure as

(1) $$\mathrm{LRE}=\ln\left(\frac{\mathrm{pv}}{\mathrm{mAs}}\right),$$

where pv is arbitrary pixel value and mAs is the tube current‐exposure time product for the related acquisition. For a given target/filter combination and kV, we generate calibration curves as a function of height (thickness) using BTE phantoms for adipose and glandular tissue referred to as R_a_(t) and R_g_(t), respectively, where t is measured in cm. These are acquired with a reference mAs_r_ = 160. For the same acquisition technique (a given target/filter combination and kV), calibration at the pixel level is given by

(2) $$pv_{\mathrm{cal}}=\frac{\mathrm{LRE}-R_{a}\left(h\right)}{R_{g}\left(h\right)-R_{a}\left(h\right)}\times 100,$$

where h is the estimated compressed breast thickness at the pixel location and LRE was calculated with Eq. (1) using the acquisition mAs. For all applications, calibration was performed at the pixel level. When considering regions of interest (ROI), results are provided as the average over the ROI or within the applicable FOV. The calibrated measure for a given mammogram is defined as the mean taken over the breast FOV referred to as μ.

Calibration methods used in the report incorporate improvements relative to earlier work. Previously, interpolation was required for the kV acquisition variable due to sparse sampling, which was eliminated in this current work due to improved sampling.^23^ A serial updating scheme is used to ensure that the baseline BTE reference data collected in the past are current. This approach permits updating the baseline reference data serially when calibration accuracy slips beyond tolerance.^24^ This monitoring involves acquiring a minimal number of BTE phantom images biweekly for each unit.

### 2.C. Correction techniques

The correction development required several technical steps. An overview is provided in this section followed by a description of each step. A thickness correction is required for each paddle on each unit.^17^, ^19^ Each paddle was characterized under stress as a function of compression force. A deformable breast biopsy practice phantom (Stereotactic needle biopsy training phantom model 013, Computerized Imaging Reference Systems, Inc., Norfolk, VA USA), referenced as DP in this report, was used to offer pliable resistance to the compression paddle. To the degree that this phantom matches the mechanical properties of the actual breast or is applicable to a wide range of breast sizes will be assessed indirectly by the correction influences on the data. In this report, we assume the paddle is a tilted plane, neglecting warp. The thickness correction is based on assuming the paddle tilts in a specific manner when a given force is applied regardless of the origin of the associated equal reactionary force supplied by the breast. Under this assumption, two breasts with differing composition compressed with the same force will induce the same paddle tilt. These assumptions apply when the breast is centrally located within the FOV. Our design goal was to develop an approximate correction that can be monitored serially with imaging. A one‐component calibration system was developed for the homogenous filler of the DP to evaluate the thickness correction measurements, representing a simplification of a two‐component calibration system developed previously to address this correction.^14^ The Cumulative Sum (Cusum) technique^25^, ^26^, ^27^, ^28^ was incorporated into this work to evaluate the seral stability of the thickness correction measurements by evaluating the accuracy of calibrated DP data. The adipose attenuation anomaly was addressed with mammograms. To adjust the adipose calibration data, image regions that approximated 100% adipose tissue (low breast density regions) were evaluated with an optimization procedure. Right and left breast calibration agreement was used to evaluate the corrections.

**Figure 2:**
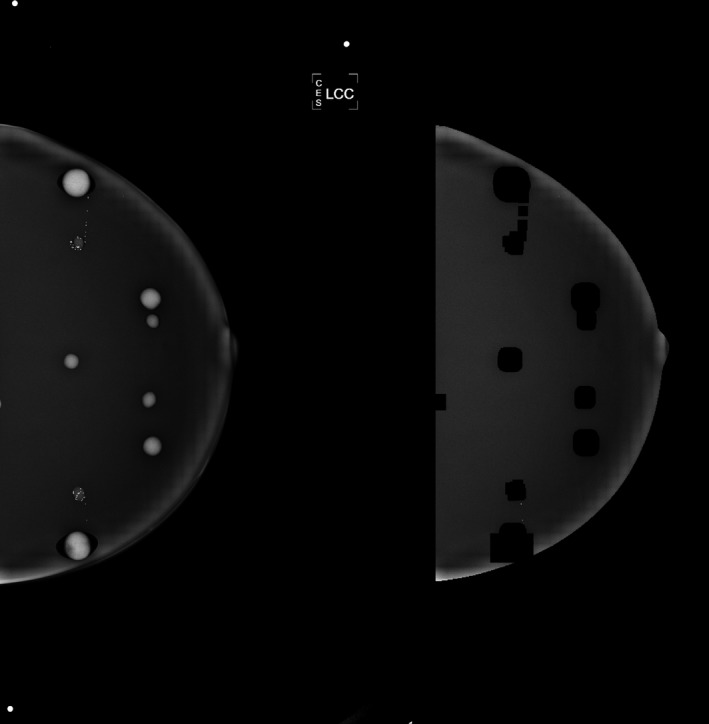
Deformable Breast Biopsy Practice Phantom (DP) Image: The DP image is shown in the left CC orientation. The image on the left shows the three reference points used for the thickness measurements: three light circular regions outside of the phantom FOV (NW, NE, and SW). The image on the right shows an example of the automated segmentation of the mass‐like features. For illustration purposes, the associated for presentation images (i.e., the related images used for clinical purposes) are used in this graphic.

The DP was used to develop the thickness corrections. Its placement on the breast support surface is shown in Fig. 1 and its corresponding image in Fig. 2 (left). If we approximate the projection of the DP as a semicircle, its radius is approximately 8–10 cm and the thickness varies in the central region from 4.5 to 5.5 cm, estimated in the relaxed position_._ The DP has a cusp on the outer edge (transition line) that is approximately 1–2 cm wide, which was excluded from the FOV. The DP was placed at the center of the breast support surface with the posterior edge of the paddle resting on its transition line (Fig. 1). It was positioned by estimating the center of the detector to mirror the procedure when patients are positioned and imaged. This phantom has mass‐type features suspended within a homogenous background comprised of a deformable but noncompressible material (Fig. 2). Paddle tilt was measured as a function of force for all paddles and units. The DP was compressed and the distance between the compression paddle and breast support surface was measured for three points for each force listed in Table 1. The perpendicular distance between the point at (x, y) of the breast support surface and the compression paddle defined the compressed breast thickness, z, measured in cm. For each DP image acquisition, self‐adhesive nipple markers were attached to the upper side of the compression paddle to identify three reference points marking the thickness measurement locations. These markers cause under exposed regions in the image enabling their detection with little uncertainty, shown in Fig. 2 (left). The placement of these markers varied about these locations. The compression paddle was lowered to make contact with the DP and an initial zero‐force reading of the system height was recorded at the reference points. These systems did not provide a force reading different from zero until approximately 10 lb is reached. Forces ranging from 10 to 35 lb were applied sequentially. A digital caliper (Marathon Watch Company LTD, Richmond Hill, ON, Canada) was used to measure z for the first two reference points. A feeler gauge method^14^ was used to measure z for the third reference point because this position was not accessible with the caliper. For each force, two image acquisitions were taken for each paddle for measurement replication, resulting in a dataset of 28 images. Three datasets per unit were collected over a 3‐month period to construct the correction surfaces. All DP images were acquired with a W/Rh combination at 26 kV on H1 and H2 and a Mo/Mo combination at 28 kV on H3. On all units, images were acquired with 160 mAs.

**Table 1:** Correction plane parameters

Force (lb)	Large paddle				Small paddle			
	a	b	c	d	a	b	c	d
H1								
0	9.543	−1.413	170.4	−115.2	9.753	−0.949	145.7	−94.53
10	8.931	−1.024	170.2	−95.67	9.695	−0.546	145.6	−74.86
15	9.968	−1.416	167.4	−67.07	9.415	−0.569	143.3	−52.99
20	16.26	−1.530	165.0	19.43	18.50	−1.011	141.4	27.60
25	16.92	−1.200	163.2	60.05	18.67	−0.554	139.6	56.52
30	20.70	−1.321	161.4	111.6	20.70	−1.015	138.6	83.46
35	22.47	−1.856	160.1	125.4	21.23	−1.080	137.8	104.7
H2								
0	9.319	−0.526	172.1	−82.11	10.13	−0.399	164.2	−76.26
10	10.49	−0.499	171.7	−69.01	11.28	−1.030	163.5	−82.09
15	9.945	−0.927	169.6	−42.35	11.05	0.006	161.6	−31.59
20	15.37	−0.075	167.8	35.60	17.08	−0.491	159.8	30.33
25	14.07	0.904	166.4	69.56	18.63	−0.503	158.2	55.49
30	20.32	0.220	165.0	112.5	22.51	−0.381	156.9	110.7
35	21.30	0.364	163.9	143.5	23.48	−0.167	156.0	130.9
H3								
0	9.295	−0.824	136.3	−70.58	10.26	−0.918	125.5	−14.23
10	9.913	−0.694	136.1	−53.04	8.508	0.205	125.3	4.324
15	9.072	0.068	134.4	9.178	11.98	−0.142	123.8	33.58
20	16.88	−0.059	133.2	56.18	16.53	−0.610	122.5	62.20
25	14.90	−0.071	132.3	52.51	14.67	−0.201	120.7	80.87
30	16.83	−0.131	131.4	80.89	17.13	0.112	112.6	112.4
35	16.50	0.147	129.4	91.44	16.67	−0.151	108.7	117.8

Correction plane parameters are provided for all units and paddles corresponding to the forces. These parameters were used to derive the relative correction surfaces.

The thickness correction was based on fitting the three measured reference points for a given force to a plane. The true thickness at a given force is expresses as

(3) $$T\left(F,x,y\right)=T_{s}+T_{r}\left(F,x,y\right),$$

where T_s_ is the readout (thickness), and T_r_(F,x,y) is the relative thickness correction for a given force referenced as F. We shifted the measured points for a given force to a relative measure expressed as

(4) $$z_{r}=z-T_{s}.$$

A given correction plane was fitted to the respective relative sample points expresses as

(5) $$z_{r}=T_{r}\left(F,x,y\right).$$

The corrected thickness can be estimated with T_s_ and the applied force expressed as

(6) $$T_{c}\left(F,x,y\right)=T_{s}+T_{r}\left(F,x,y\right).$$

The T_r_ (F,x,y) surface was derived by solving the standard plane equation given by

(7) $$\text{ax}+\text{by}+{\text{cz}}_{r}=d.$$

In the above equation, x and y were scaled to cm. Once establishing the relative corrections, the absolute correction was applied to a given image by reading the Force and T_s_ from the image header file and applying Eq. (6). The parameters in Eq. (7) were used to characterize and compare the correction planes. In this form, the vector normal to the correction plane can be expressed in component form as **N **= (a, b, c) and is central to the analysis, where the angles *α*,* β*, and *γ* are the respective directional angles between **N** and the unit vectors along the x, y, and z coordinate directions. For intermediate forces not cited in Table 1, we used linear interpolation to generate the plane parameters.

Two methods were used to evaluate the thickness corrections. As an initial step to show consistency, we acquired serial DP images for monitoring changes in the paddle tilt and T_s_. For each unit and paddle, 6 DP images were acquired biweekly for approximately 2 years, corresponding to the forces shown in Table 1 except for the zero force. These images were not used for the correction surface construction. Because caliper measurements require considerable effort, we monitor the correction applicability serially by checking calibration accuracy using the DP. As long as the calibrated values of the DP are in agreement with the ideal value (i.e., 100SU), the paddle characterization as a function of force is considered correct (i.e., is in agreement with the initial measurements).

For calibration purposes, the filler within the DP was treated as a one‐component system and a calibration curve was established.^23^ The background component of the DP was designated with 100SU. The calibration curve, R_DP_(t), was constructed by acquiring images over varying thicknesses for each force with 160 mAs and generating the respective LRE sample points using Eq. (1). Linear interpolation was used to generate intermediate calibration points not acquired with imaging. For this one‐component system, calibration at the pixel level is expressed as

(8) $$\mathrm{calibrated}\;\mathrm{DP}\;\mathrm{pixel}=\frac{100}{R_{\mathrm{DP}}\left(h\right)}\times \mathrm{LRE},$$

where h is the compressed DP thickness at the pixel location. The 100 ± 4SU range was used to evaluate the mean calibrated value of these regions (entire interior background excluding excluded regions shown in Fig. 2). The initial calibration curve was developed by manually segmenting the DP background regions (eliminating the anomalies). Thereafter, the background regions in the biweekly images were isolated from the simulated anomalies with an automated algorithm. This algorithm used calibration data to determine the appropriate thresholds noting that the intensities of the suspended anomalies deviate considerably from the background filler material. All segmented images were visually assessed before using them for analytical purposes. The algorithm did not experience failures over the course of this study (see Fig. 2). The DP background was calibrated with and without the thickness correction at the pixel level.

The accuracy of the characterized compression paddle for each unit was monitored serially with the Cusum technique described in our previous work^24^, ^29^ adapted to DP images. Briefly, the Cusum argument is expressed as

(9) $$U_{n}=\frac{m_{n-}m_{0}}{\left|m_{0}\right|},$$

for n > 0 and U_0_ = 0, where m_0_ and m_n_ are the LREs for the DP taken at time = 0 and at serial time points referenced as n measured in days. The standard sequential Cusum is expressed as

(10) $$S_{n}=\sum_{i=1}^{n}S_{n-1}+U_{n}.$$

In practice, we use the decision interval (DI) from of the Cusum to monitor and detect sustained drift beyond our predetermined tolerance. Upward drift is monitored with this DI form

(11) $$S_{n}^{+}=\max\left(0,S_{n-1}^{+}+U_{n}-k\right)$$

and downward by this form

(12) $$S_{n}^{-}=\min\left(0,S_{n-1}^{+}+U_{n}+k\right),$$

where k is the chart constant with $S_{0}^{+}=S_{0}^{-}=0$ **.** We used an empirically defined tolerance described previously^23^, ^24^ of ±4SU for two consecutive serial points, which applies to reference phantom images. We note, k was adjusted for given acquisition (i.e., kV, target/filter combination, thickness) to detect the same tolerance ±4SU.^24^ When the deviation at any time point is less than the tolerance, the DI terms return zero, indicating in control behavior. When the calibration accuracy drifts beyond the established tolerance for a given unit, the paddle is recharacterized. The Cusum is then reset and the monitoring is re‐established.

For the second thickness correction evaluation, we investigated the similarity between left and right calibrated CC mammograms under the premise that the correction should improve the agreement in μ. This included analyzing the entire sample and splitting it into two subgroups. One subgroup included women who had different acquisition techniques between left and right views defined by meeting at least one of these criteria: different kV, different target‐filter, compressed breast thickness system readout differences of at least 5 mm, or differing compression paddles. The other subgroup included women who had the same acquisition technique for left and right views defined by not meeting the above criteria. Right and left breast calibration agreement was characterized with Bland–Altman plots.^30^ In this approach, the right and left breast difference in μ, defined as μ_d_, was plotted against the right and left breast average of the respective μ defined as μ_a_. To complement these plots, the mean and standard deviations of the relevant noncorrected and corrected distributions were characterized with 95% confidence intervals. These quantities include: the mean and standard deviation of the μ_a_ distribution defined as m_a_ and *σ*_a_, respectively; and the mean and standard deviation of the μ_d_ distribution defined as m_d_ and *σ*_d_, respectively. The variances of the μ_d_ distributions were evaluated before and after the corrections and were compared with an F‐test.

### 2.D. Attenuation anomaly

The x‐ray attenuation of the 100% adipose BTE material used for establishing calibration references is greater than that of actual adipose breast tissue.^11^ To detect the presence of this anomaly, calibrated mammograms were analyzed in restricted regions where the compressed breast thickness uncertainty was minimal. For a given mammogram and force, this region was defined within a strip (35 pixels in width) between the reference points near the posterior edge of the compression paddle. This strip was searched automatically with a 35 × 35 pixel window. A calibrated value (average taken over all pixels within the window) well below zero is an indication the anomaly exists when the compressed thickness is known, noting that a calibrated value near zero indicates 100% adipose tissue. When present, the respective 35 × 35 pixel ROI was marked for this analysis. Ten mammograms for each target/filter combination were used for this analysis. These mammograms were not used in the evaluation studies. In this analysis, we neglect influences due to varying kV for a given target/filter. This idealization assumes the correction factor can be approximated as a constant for fixed target/filter. This is based on these approximations: (a) the attenuation coefficient for adipose tissue varies slowly with respect to a change in kV for fixed target/filter in comparison to a change in target/filter for fixed kV, (b) the magnitude of the correction factor is much less than the attenuation for adipose tissue over the entire range of acquisition settings, and (c) the variation in kV is mainly captured by the adipose uncorrected calibration data. We note, we used a similar approximation previously when developing calibration methods for another FFDM technology.^11^ For reference, the effective x‐ray attenuation coefficients for these Selenia units were presented in our related work.^23^ The correction factor was used to modify the respective adipose calibration curve expressed as

(13) $$R_{a}^{c}\left(t\right)=R_{a}\left(t\right)+\Delta \times t,$$

where Δ is the respective correction factor and the superscript indicates the corrected curve. Differential evolution optimization^31^ was used to estimate the correction factors for each target/filter combination using Eq. (13). Calibration was performed using the corrected adipose expression in Eq. (2). The correction factor that minimized the absolute difference between the calibrated value and the ideal value (zero) for all ROIs was used as the respective correction for a given target/filter combination. To evaluate this correction, it was applied in tandem with the thickness correction using Bland–Altman plots and the related analyses discussed above.

## 3 Results

### 3.A. Compression paddle characterization

To a close approximation, all paddles showed common behavior with tilt along the same direction. This is noted by the plane coefficients shown in Table 1, where the coefficient for y (i.e., b) is much smaller than the other coefficients (i.e., a or c) for all paddles and forces. The validity of this approximation is noted by considering the angle, **N **= (a, b, c), made with the unit vectors along the y and z coordinate directions. As shown in Table 2, *β* changed little as a function of force and was approximately 90 degrees. This indicated **N,** to a good approximation, is constrained to the xz plane because the front and back edges of all paddles were parallel to the breast support surface for all forces. In contrast, *γ* (the angle that measures the tilt from the z axis is in the xz plane) changed slowly as a function of force (approximately within 3°–9°). All paddles showed a similar trend in that the deflection angle increased upward from the horizontal plane; the deflection angle is approximately *α *= 90 ‐ *γ* in the xz plane as force increase. All paddles had similar values in *γ* per force when considering the associated uncertainty, provided parenthetically in Table 2. The differences in the paddles are noted by the constant d. As d approaches zero, the difference between z_r_ and T_s_ approached zero at x = 0 and y = 0, noting that the z_r_ intercept is given by d/c.

**Table 2:** Correction plane directional angles

Force (lb)	H1				H2				H3			
	Large paddle		Small paddle		Large paddle		Small paddle		Large paddle		Small paddle	
	*γ*	*β*	*γ*	*β*	*γ*	*β*	*γ*	*β*	*γ*	*β*	*γ*	*β*
0	3.2(0.2)	90.5	8.8(0.2)	90.4	3.1(0.2)	90.2	3.5(0.3)	90.1	3.9(0.1)	90.4	4.7(0.3)	90.4
10	3.0(0.1)	90.3	3.8(0.2)	90.2	3.5(0.1)	90.2	4.0(0.1)	90.4	4.2(0.1)	90.3	3.9(0.6)	89.9
15	3.4(0.7)	90.5	3.8(0.5)	90.2	3.4(0.4)	90.3	4.0(0.4)	90.0	3.9(0.8)	90.0	5.5(0.8)	90.1
20	5.7(0.5)	90.5	7.4(0.5)	90.4	5.2(0.2)	90.0	6.1(0.1)	90.2	7.2(0.1)	90.0	7.7(0.2)	90.3
25	5.9(0.8)	90.4	7.6(0.6)	90.2	4.8(0.4)	89.7	6.7(0.4)	90.2	6.4(0.4)	90.0	6.9(0.6)	90.1
30	7.3(0.6)	90.5	8.5(0.3)	90.4	7.0(0.2)	89.9	8.2(0.2)	90.1	8.2(0.2)	90.1	8.6(0.5)	89.9
35	8.0(0.5)	90.7	8.8(0.2)	90.4	7.4(0.4)	89.9	8.5(0.1)	90.1	8.6(0.1)	89.9	8.7(0.5)	90.1

This shows the relevant directional angles (degrees) as a function of compression force for the normal vector **N** for all paddles. To a good approximation, *α *= 90 ‐ *γ* because **N** is constrained to the xz plane. The estimated uncertainty in *γ* is provided parenthetically.

**Figure 3:**
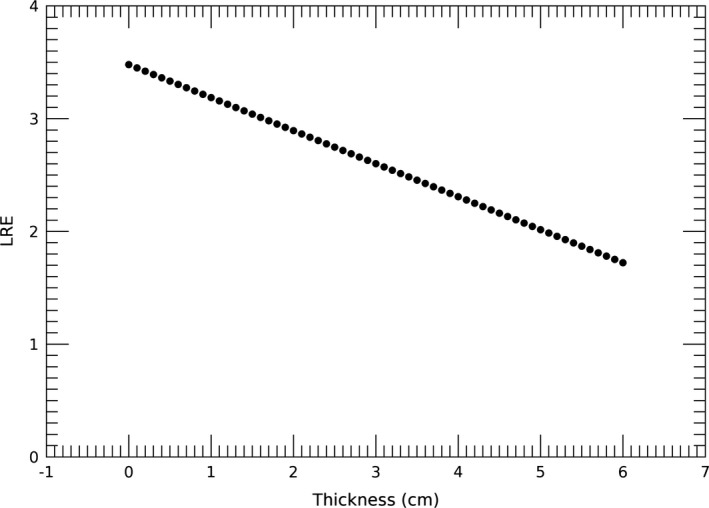
Deformable Breast Biopsy Practice Phantom (DP) Calibration Curve: This shows the logarithmic relative response (LRE) calibration curve for the DP phantom as a function of compressed thickness measured in cm. The points were generated using repeated measurements of the sampled forces. The fine spacing is a consequence of interpolation.

**Figure 4:**
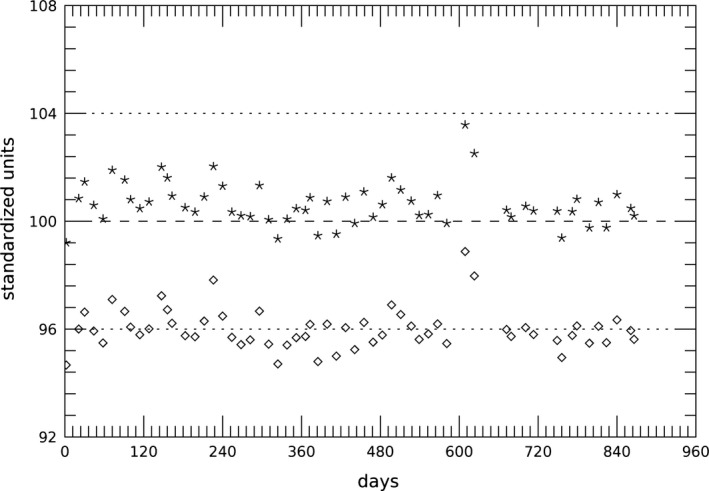
Serial Calibration accuracy: The deformable breast biopsy practice phantom was imaged serially and calibrated without the thickness correction (diamonds) and with the thickness correction (stars). This example is from H2 with force = 35 lb. The central dashed line represents the ideal standardized unit (SU) =100, whereas the upper and lower dashed lines mark the ± 4SU tolerance.

Serial DP images were calibrated as the first step of the thickness correction evaluation. The related calibration curve is shown in Fig. 3. We used H2 with force = 35 lb to exemplify the findings by comparing the calibration with and without the correction illustrated in Fig. 4. This shows that the correction produced a calibration accuracy within the ±4SU tolerance from the ideal value = 100SU for the DP, whereas many of the uncorrected points are outside of this tolerance. The corresponding DI Cusum serial monitoring returned zero for Eqs. (11) and (12) over the relevant timeframe for all units and forces indicating the corrections were stable over this interval (plots not shown).

### 3.B. Attenuation anomaly

The attenuation anomaly findings are discussed first because they were used in the combined correction evaluation with calibrated mammograms. A correction was estimated for the Mo/Mo, Mo/Rh, W/Rh, and W/Ag beams. Without correction, averaging of the ROIs for each respective beam gave: −12.9, −13.3, −13.9, and −9.50. For each beam, the respective estimated effective x‐ray attenuation coefficient (cm^−1^) correction, Δ, was 0.024, 0.020, 0.024, and 0.016. The corrected calibrated ROI averages were −0.83, −1.1, −0.79, and 0.53, respectively. These were within ±4SU from the ideal value (zero) for adipose tissue.

### 3.C. Correction evaluation

**Figure 5:**
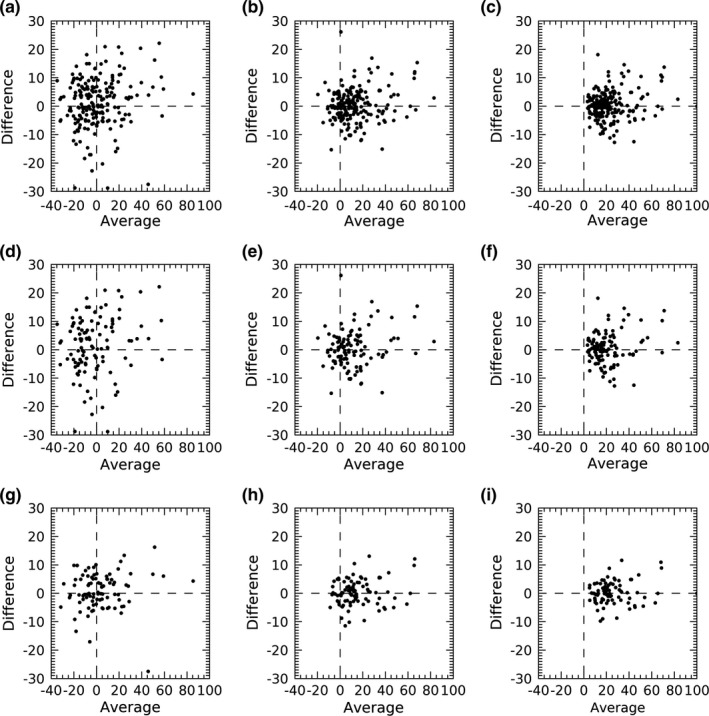
Bland–Altman Plots. The left and right breast difference (μ_d_) is plotted on the vertical axes and the left and right breast average (μ_a_) on the horizontal axes. Plots are shown for the noncorrected (left), thickness corrected (middle), and combined thickness and attenuation anomaly corrected (right) data. Plots in the top row include the entire dataset (a–c). The middle row shows the plots for the subgroup with different acquisition techniques (d–f), and the bottom row shows the plots for the subgroup with the same acquisition techniques (h–i).

Bland–Altman plots are shown in Fig. 5. The top row shows plots for the entire dataset, which are discussed first. The impact of thickness correction is noted by comparing the uncorrected data (a) with the corrected data (b) in Fig. 5. The associated descriptive metrics are provided in Table 3. The uncorrected data were clustered around μ_a_ = 0, where approximately 57% of the samples were negative. The mean of the noncorrected data (m_a_) was not significantly different from zero, whereas the thickness corrected mean (m_a_ = 13.99) was significantly greater than zero, while noting that approximately 23% of μ_a_ samples remained negative. The μ_d_ distribution mean (m_d_) was not significantly different from zero in either the noncorrected or thickness‐corrected data indicating a lack of right‐left breast bias in μ. The linear correlation between right and left μ increased from R = 0.90–0.96 (see Table 3) due to the correction. The F‐test results, provided in Table 4, showed that the μ_d_ distribution variance was reduced significantly (*P* < 0.0001), noted by comparing the respective *σ*_d_ in Table 3. When applying both corrections in tandem (c), the μ_a_ distribution mean shifted significantly in the positive direction with m_a_ = 22.8 relative to applying the thickness correction in isolation, noting one sample had negative μ_a_. When applying both corrections in tandem, *σ*_d_ was not significantly influenced relative to applying the thickness correction in isolation (5.81 vs 5.32) and R was unchanged. The middle and bottom rows of Fig. 5 show the findings from the subgroups with different and the same acquisition techniques, respectively. These subgroups exhibited similar behavior noting m_a_ was not significantly different from zero prior to applying the corrections; 61% and 50% of the samples in (d) and (g), respectively, were negative. After applying the thickness correction approximately 26% percent of the samples shown in (e) and 16% shown in (i) remained negative. In general, the corrections had a similar influence on both subgroups where m_a_ experienced significant shifts to the right after each correction with m_d_ not significantly different from zero. The thickness correction reduced the variance in the μ_d_ distribution in both subgroups significantly (Table 4). When applying both corrections in tandem, one of the samples remained negative in the different acquisition subgroup while no samples were negative in the same acquisition group.

**Table 3:** Descriptive metrics

Internal evaluation	No correction	Thickness correction	Both corrections
Full dataset (n = 199)			
R	0.90 (0.88, 0.93)	0.96 (0.88, 0.93)	0.96 (0.95, 0.97)
μ_d_	1.21 (−0.01, 2.43)	0.43 (−0.38, 1.24)	0.38 (−0.36, 1.12)
*σ*_d_	8.79 (8.00, 9.75)	5.81 (5.29, 6.45)	5.32 (4.85, 5.90)
μ_m_	0.92 (−1.77, 3.62)	13.99 (11.17, 16.80)	22.83 (20.32, 25.34)
*σ*_m_	19.41 (17.68, 21.53)	20.25 (18.44, 22.46)	18.08 (16.46, 20.02)
Different acquisition technique (n = 111)			
R	0.87 (0.83, 0.90)	0.94 (0.92, 0.95)	0.93 (0.91, 0.95)
μ_d_	1.58 (−0.33, 3.48)	0.44 (−0.79, 1.66)	0.35 (−0.78, 1.49)
*σ*_d_	10.24 (9.05, 11.80)	6.58 (5.82, 7.58)	6.10 (5.39, 7.02)
μ_m_	−1.37 (−4.92, 2.19)	11.41 (8.04, 2.19)	20.52 (17.52, 23.52)
*σ*_m_	19.10 (16.88, 22.01)	18.14 (16.03, 20.90)	16.12 (14.24, 18.57)
Same acquisition technique (n = 88)			
R	0.95 (0.93, 0.96)	0.98 (0.97, 0.98)	0.98 (0.97, 0.98)
μ_d_	0.74 (−0.62, 2.11)	0.42 (−0.57, 1.40)	0.41 (−0.46, 1.28)
*σ*_d_	6.54 (5.70, 7.68)	4.70 (4.09, 5.52)	4.18 (3.64, 4.91)
μ_m_	3.81 (−0.27, 7.89)	17.24 (12.57, 21.90)	25.74 (21.57, 29.92)
*σ*_m_	19.53 (17.01, 22.93)	22.31 (19.43, 26.20)	19.99 (17.41, 23.47)

This table gives descriptive metrics for the non‐corrected, thickness corrected, and combined thickness and attenuation anomaly corrected data. These include the right and left breast linear correlation coefficient (R), the right and left breast difference (μ_d_) distribution mean (m_d_) and standard deviation (*σ*_d_), right and left breast average distribution mean (m_a_), and standard deviation (*σ*_a_). Each quantity is provided with 95% confidence intervals.

**Table 4:** Variation comparisons

F‐test comparing μ_d_ distributions *P*‐value	Full dataset (n = 199)	Different acquisition technique (n = 111)	Same acquisition technique (n = 88)
No correction vs thickness correction	<0.0001	<0.0001	0.0023
No correction vs both corrections	<0.0001	<0.0001	<0.0001

The variance from the right and left breast calibrated difference distributions were compared within each group. This table gives the *P*‐values from the F‐test. The noncorrected data were used as the reference.

## 4 Discussion

We investigated two technical challenges noted previously when developing a phantom‐based calibration method for FFDM. A compressed breast thickness correction methodology built on earlier work was presented in this report. The adipose attenuation anomaly correction was also evaluated. Our findings, both past and present, indicate that a calibration approach based on references derived from BTE phantom imaging requires a compressed breast thickness correction tailored to the specific unit and paddle. The thickness correction merits are noted by μ_a_ distribution shift in the positive direction, the elimination of negative μ, and the variance reduction of the μ_d_ distribution. Our findings also indicate the attenuation anomaly correction was required as noted by the corresponding μ_a_ distribution shift in the positive direction, in agreement with our previous work.^11^ The two corrections applied in tandem eliminated negative μ substantially. In total, this evidence indicates that the DP is suitable for characterizing the compression paddle tilt and offset for H units as demonstrated with the large and small paddle findings. Our approach includes an initial paddle evaluation with physical measurements followed by serial monitoring using calibration accuracy as the quality endpoint. The serial monitoring is based acquiring DP images biweekly for each unit and paddle. The present report provides an internal validation of the corrections using calibration accuracy as the endpoint.

Our thickness correction approach is similar to that of several studies in that direct measurements of the perpendicular distance between the breast support surface and the compression paddle surface at multiple (x, y) locations were used to establish the correction as a function of compression force.^17^, ^18^, ^19^ Photogrammetry techniques have also been studied using both phantoms and mammograms.^17^, ^18^ Image processing techniques based on tissue characteristics and gray‐level distributions^20^, ^21^ have also been investigated. One main difference with our methodology is that we continually monitor the thickness correction measurements serially using calibration accuracy as the endpoint.

There are several qualifications with our study and findings worth noting. The absolute truth for the LCC‐RCC breast density measurement similarity is unknown leaving the relative agreement as the standard. The DP volume (or size) is fixed, whereas breast volumes vary considerably. Our thickness correction did not take breast volume into consideration explicitly. Implicitly, breast volume was partially accounted for by the paddle selection. We have also made the assumption that the resistance of the DP matches the resistance offered by actual breast. Our findings indicate these are reasonable approximations. These findings are relevant to the specific type of calibrated measurement used in this analysis (the mean). As noted, we have developed other calibrated metrics. We only considered CC views. Both the correction and calibration methods apply to mediolateral oblique and mediolateral views but would require additional processing to detect the pectoral muscle region so it could be removed from the calibration measurement calculation.

## 5 Conclusion

This work provided insight into the technical nuances of developing a general calibration system intended to apply across FFDM technologies using references derived from phantom imaging. The validation of the calibration methodology and the corrections will require further evaluation using defined experimental endpoints such as breast cancer status or comparisons with other breast density measures. Further testing, for example, would include evaluating whether it is possible to merge calibrated data from different technologies within the context of a case–control study without introducing bias relative to the type of units used to acquire the mammograms, which is in process. We also note that mammography is shifting to digital breast tomosynthesis (DBT). DBT volumetric data may be difficult to calibrate with these methods due to the reconstruction processing. However, calibration applications are still relevant for DBT. The 2D projection images used for the DBT volume reconstruction can be calibrated in theory. Additionally, the combo‐mode acquisition is also used for screening and includes standard 2D FFDM images, which can be calibrated.

## Conflicts of interest

The authors have nothing to disclose.
